# Cardiovascular Emergencies

**DOI:** 10.1155/2017/7210261

**Published:** 2017-07-02

**Authors:** Yu-Jun Chang, Shih-Lin Chang, Eric Chong, Kazuyoshi Suenari, Antonios Michalopoulos

**Affiliations:** ^1^Epidemiology and Biostatistics Center, Changhua Christian Hospital, Changhua, Taiwan; ^2^Division of Cardiology, Taipei Veterans General Hospital, Taipei, Taiwan; ^3^Division of Cardiology, Ng Teng Fong General Hospital, Singapore; ^4^Department of Cardiovascular Medicine, Hiroshima University Graduate School of Biomedical Sciences, Hiroshima, Japan; ^5^1st Propedeutic Surgical Department, AHEPA University Hospital, Medical School, Aristotle University of Thessaloniki, Thessaloniki, Greece

Cardiovascular disease is the most prevalent disease worldwide. It is the leading global cause of death, accounting for 15 million deaths in 2015 [1]. Cardiovascular disease often presents in emergency situations; prompt treatment is essential to reduce mortality. “Time is gold” has always been the cornerstone of cardiovascular emergency management, as every 30-minute delay in door to balloon time translates into 7.5% relative increase in mortality [2]. Rapid evolution in knowledge, technology, and healthcare system efficiency play important roles in saving patients' lives during cardiovascular emergencies. The common treatment goal is to shorten the door to balloon time; rapid ambulance transfer service, early emergency department triage, and seamless team work are key factors in lowering the door to balloon time to new limit.

Today, acute ST elevation myocardial infarction, cardiogenic shock, and out-of-hospital cardiac arrest are the most life-threatening cardiovascular emergencies. Many attempts have been made to improve the healthcare system and efficacy to lower the mortality further. In this special issue, internationally renowned authors shared their invaluable research in management of cardiac arrest, acute myocardial infarction, and cardiogenic shock. The data on prognostic analysis of cardiogenic shock during acute myocardial infarction are being presented. The data and advantage of further reduction in door to balloon time during primary coronary intervention to less than 60 min compared to over 60–90 min are being addressed. The public knowledge of bystander cardiopulmonary resuscitation and, in particular, the nurse's role for saving on-scene time for out-of-hospital collapse are being examined in detail. Further public awareness campaign and hospital workflow can be proposed based on these valuable data.

Out-of-hospital cardiac arrest is one of the most dreadful conditions leading to over 90% mortality rate [3]; for patients being resuscitated on time and restoring spontaneous circulation, many develop cardiogenic shock requiring inotropic and mechanical support beyond emergency revascularization. Aggressive adjunctive treatments are required to promptly correct hypoxia, normalize acidosis and electrolytes imbalance, improve cardiac output, and revert multiorgan failure. Cooling therapy plays additional role in reducing hypoxic brain injury [4]. In this special issue, novel treatment strategies including the utilization of glutamine to protect against postarrest acidosis are being discussed. Invaluable experience on hemodynamic analysis of pediatric cardiogenic shock using transpulmonary thermodilution is being highlighted.

This special issue provides the readers with deeper insight into the management of high risk cardiovascular emergencies and the topics are highly interest-generating and are scientifically valid.

*Yu-Jun Chang* *Yu-Jun Chang* *Shih-Lin Chang* *Shih-Lin Chang* *Eric Chong* *Eric Chong* *Kazuyoshi Suenari* *Kazuyoshi Suenari* *Antonios Michalopoulos* *Antonios Michalopoulos*
